# Exploitation of the Host Ubiquitin System: Means by *Legionella pneumophila*

**DOI:** 10.3389/fmicb.2021.790442

**Published:** 2021-12-22

**Authors:** Jingjing Luo, Lidong Wang, Lei Song, Zhao-Qing Luo

**Affiliations:** ^1^Department of Respiratory Medicine, Center for Pathogen Biology and Infectious Diseases, Key Laboratory of Organ Regeneration and Transplantation of the Ministry of Education, The First Hospital of Jilin University, Changchun, China; ^2^Department of Biological Sciences, Purdue University, West Lafayette, IN, United States

**Keywords:** ubiquitin, intracellular pathogen, effector, immunity, virulence

## Abstract

Ubiquitination is a commonly used post-translational modification (PTM) in eukaryotic cells, which regulates a wide variety of cellular processes, such as differentiation, apoptosis, cell cycle, and immunity. Because of its essential role in immunity, the ubiquitin network is a common target of infectious agents, which have evolved various effective strategies to hijack and co-opt ubiquitin signaling for their benefit. The intracellular pathogen *Legionella pneumophila* represents one such example; it utilizes a large cohort of virulence factors called effectors to modulate diverse cellular processes, resulting in the formation a compartment called the Legionella-containing vacuole (LCV) that supports its replication. Many of these effectors function to re-orchestrate ubiquitin signaling with distinct biochemical activities. In this review, we highlight recent progress in the mechanism of action of *L. pneumophila* effectors involved in ubiquitination and discuss their roles in bacterial virulence and host cell biology.

## Introduction

**FIGURE 1:**
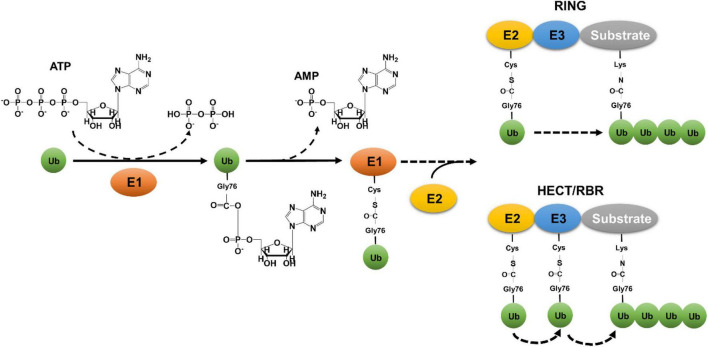
Canonical eukaryotic ubiquitination by the three-enzyme cascade. Canonical ubiquitination is initiated by E1-mediated acyl adenylation on the carboxyl terminus of Ub by hydrolyzing an ATP molecule, followed by a linkage of Ub to the active cysteine residue of E1 and the release of AMP. The Ub on E1 is then delivered to the active cysteine residue of E2, which works together with E3 to transfer Ub to lysine residue of substrates. E3 ligases are categorized into three different classes: RING (Really Interesting New Gene) type, HECT (Homologous to the E6-AP Carboxyl Terminus) type, and the RBR (RING-between-RING) type. While the RING domain-containing E3 ligases transfer ubiquitin from the thioester-conjugated E2 directly to the isopeptide bond of lysine residues in the substrates, HECT- or RBR-containing E3 enzymes receives ubiquitin on a cysteine residue from E2, which is then delivered to substrate proteins.

Ubiquitin (Ub) is a small, 76-amino acid protein that highly conserved in all eukaryotes (Liu et al., 2013; Shaid et al., 2013). The attachment of ubiquitin to client proteins forms a covalent modification known as ubiquitination, resulting in changes in the stability, cellular localization and/or activity of the substrates. Classical ubiquitination is a three-reaction cascade catalyzed by three types of enzymes (Figure 1). The first reaction is carried out by the ubiquitin-activating enzyme (E1), which catalyzes acyl adenylation on the carboxyl terminus of Ub by hydrolyzing an ATP molecule. The labile intermediate AMP-Ub then reacts with E1, resulting in linkage of the Ub moiety to its active cysteine residue by a thioester bond and the release of AMP (Hershko and Ciechanover, 1998). The Ub on E1 is then delivered to the active cysteine residue of ubiquitin-conjugating enzyme (E2) (Olsen and Lima, 2013). Charged E2s carrying a Ub moiety work together with ubiquitin ligation enzymes (E3) to transfer the modifier onto a substrate (Buetow and Huang, 2016). Whereas E2s often dictate the chain type of ubiquitination (Stewart et al., 2016), E3s normally determine substrate specificity (Buetow and Huang, 2016). There are three major classes of E3 enzymes that utilize distinct mechanisms to transfer Ub from E2 or E3 itself to the substrate (Buetow and Huang, 2016). In most cases, Ub is attached to substrates by an isopeptide bond formed between its carboxyl terminus and the ε-amino group of Lys residues in target protein or in the preceding Ub moiety (Hershko and Ciechanover, 1998).

Ubiquitination can be reversed by deubiquitinases (Dubs), which are a large family of enzymes that cleave the isopeptide bond between Ub and its substrate or between Ub moieties, leading to the release of free Ub and the return of the substrate protein to its original state (Sowa et al., 2009). Together with E3 ligases, Dubs regulate a wide variety of cellular processes, such as apoptosis, cell cycle and division, mutation repair, transcription, differentiation, development, inflammation and immune responses (Hershko and Ciechanover, 1998). Under certain disease and development conditions, ubiquitin signaling is regulated by posttranslational modifications on one or more of the enzymes or Ub itself, which allow the cell to fine-tune specific signal transduction cascades regulated by ubiquitination (Song and Luo, 2019). Furthermore, a recent exciting discovery demonstrates that the lipid A moiety of bacterial lipopolysaccharide (LPS) is ubiquitinated by the E3 ubiquitin ligase RNF213, an event critical for the elimination of invading pathogenic *Salmonella* Typhimurium (Otten et al., 2021). The identification of non-proteinaceous substrates has greatly expanded the scope of the biological processes regulated by this posttranslational modification.

Because of the involvement of ubiquitination in diverse cellular processes, particularly in immunity, it is fulfilling to realize that many pathogens elaborate various pathogenic factors to co-opt ubiquitin signaling to facilitate their survival and replication in their hosts. Virulence factors involved in interference of the host ubiquitin network have been identified in a wide spectrum of pathogens (Zhou and Zhu, 2015; Vozandychova et al., 2021). Among these, the opportunistic pathogen *Legionella pneumophila* is emerging as a unique model in this regard because of the sheer number of bacterial proteins involved and the richness in their mechanisms of action (Qiu and Luo, 2017b).

*L. pneumophila* is a Gram-negative bacterium ubiquitously found in fresh water environments where it exists as a parasite for protists. Inhalation of bacteria-contaminated aerosol by susceptible individuals can lead to the development of a severe form of pneumonia called Legionnaires’ disease (Newton et al., 2010). *L. pneumophila* employs similar strategies to survive and replicate in amoebae and human macrophages, in both cases, it utilizes the Dot/Icm type IV secretion system to transport more than 330 effector proteins into host cells to construct the *Legionella*-containing vacuole (LCV) supportive for bacterial growth (Swanson and Isberg, 1995; Finsel and Hilbi, 2015; Ensminger, 2016). The LCV highly resembles the endoplasmic reticulum (ER) in term of protein composition and other cell biological features (Swanson and Isberg, 1995; Kagan and Roy, 2002). These Dot/Icm effectors interfere with a wide range of host processes with diverse biochemical activities (Qiu and Luo, 2017b). Among them, close to 20 effectors have been found to co-opt the host ubiquitin network by diverse biochemical mechanisms (Table 1; Qiu and Luo, 2017a). Here, we will highlight recent progress in the study of *Legionella* proteins that hijack host ubiquitination and their roles in bacterial pathogenesis and in the study of host cell biology.

**TABLE 1:** *L. pneumophila* Dot/Icm effectors involved in ubiquitination pathway.

Effectors (Gene ID)	Aliases	Interactor/substrate	Enzymatic activity	Function	References
**Canonical E3 ligases**					
lpg0171	legU1	SKP1, Cullin 1, BAT3	F-Box protein, E3 ligase	Unknown	Ensminger and Isberg, 2010; Skaar et al., 2013
lpg1111	RavN	Unknown	U-box-like protein, E3 ligase	Unknown	Lin et al., 2018
lpg1408	licA	SKP1	F-Box protein	Unknown	Ensminger and Isberg, 2010
lpg2144/lpp2082	legAU13/ankB	SKP1, Cullin 1, Parvin B	F-Box protein, E3 ligase	Intracellular replication; Recruitment of ubiquitinated proteins to the LCV	Al-Khodor et al., 2008; Price et al., 2009; Ensminger and Isberg, 2010; Lomma et al., 2010; Skaar et al., 2013
lpg2224	PpgA	Unknown	F-Box protein	Unknown	Ensminger and Isberg, 2010
lpg2370	HipA	Unknown	E3 ligase	Unknown	Lin et al., 2018
lpg2455	GobX	Unknown	U-Box protein, E3 ligase	Unknown	Lin et al., 2015
lpg2498	MavJ	Unknown	E3 ligase	Unknown	Lin et al., 2018
lpg2525		Unknown	F-Box protein	Unknown	
lpg2577	MavM	Unknown	E3 ligase	Unknown	Lin et al., 2018
lpg2830	LegU2/LubX	Clk1, SidH	U-Box protein, E3 ligase	SidH degradation	Luo and Isberg, 2004; Kubori et al., 2008, 2010; Quaile et al., 2015
**SidC family**					
lpg2452	LegA14/SdcB	Unknown	Cys-His-Asp domain protein, E3 ligase	Unknown	Lin et al., 2018
lpg2510 lpg2511	SidC SdcA	Rab10	Cys-His-Asp domain protein, E3 ligase	Recruitment of ER vesicles and ubiquitinated proteins including Rab10 to LCV	Weber et al., 2006; Ragaz et al., 2008; Hsu et al., 2014; Luo et al., 2015; Jeng et al., 2019
**Non-canonical E3 ligases**					
lpg0234 lpg2153 lpg2156 lpg2157	SidE SdeC SdeB SdeA	Rab1, Rab6a, Rab30, Rab33b, Rtn4, FAM134C	All-in-one ubiquitin conjugation enzyme; Deubiquitinase	Intracellular replication; regulation of ubiquitin dynamics on the LCV; Recruitment of ER markers to the LCV; ER tubule Rearrangement.	Bhogaraju et al., 2016; Qiu et al., 2016; Kotewicz et al., 2017; Wan et al., 2019a; Shin et al., 2020b; Kawabata et al., 2021
lpg2147	MavC	UBE2N	Transglutaminase activity	inhibits NFκB activation	Valleau et al., 2018; Gan et al., 2019a; Guan et al., 2020; Puvar et al., 2020
**Dubs**					
lpg0160	RavD	Unknown	Cleaves linear Ub chains	Benefits L. pneumophila by suppressing host immune responses	Wan et al., 2019b
lpg0227	Ceg7	Unknown	Targets K6-, K11-, K48-, and K63- Ub chains	Unknown	Hermanns et al., 2020; Schubert et al., 2020
lpg1148	LupA	LegC3	Typical bacterial CE clan Dubs	Removes Ub modification of LegC3	Urbanus et al., 2016
lpg1621	LotB	Sec22b	Targets K63- Ub chains	Abolishes the interaction between Sec22b on the LCV and the syntaxin 3	Kitao et al., 2020b; Shin et al., 2020a
lpg2248	LotA	Unknown	Targets K13-, K48-, and K63- Ub chains	Removes ubiquitinated proteins from the surface of LCV	Kubori et al., 2018
lpg2529	LotC/Lem27	Rab10	Targets K6-, K11-, K48-, and K63- Ub chains	Removes ubiquitinated Rab10 from the surface of LCV	Liu et al., 2020; Schubert et al., 2020; Shin et al., 2020a

## Canonical E3 Ligases

The U-box type of E3s usually contain a conserved U-box sequence of 70 amino acids at their carboxyl termini (Miyamoto and Saito, 2018). LubX (Lpg2830) and GobX (Lpg2455) are *L. pneumophila* effectors that belong to this E3 family. Interestingly, LubX contains two U-box motifs with distinctly different functions: only U-box1 confers E3 activity (Quaile et al., 2015) and the proteins targeted by this enzyme appear structurally diverse. First, it attacks SidH (Lpg2829), a large Dot/Icm substrate (Luo and Isberg, 2004) encoded by a gene next to *lubX* by ubiquitination and subsequent degradation by the proteasome (Kubori et al., 2010). Because LubX functions to regulate the activity of another effector, it was designated as a metaeffector (Kubori et al., 2010). Interestingly, metaeffectors seem to be used in the regulation of a large cohort of *Legionella* effectors (Urbanus et al., 2016; Joseph and Shames, 2021; McCloskey et al., 2021). Second, LubX mediates ubiquitination of the Cdc2-like kinase 1 (CLK1) in host cells, leading to its degradation, but the biological significance of this degradation remains unknown (Kubori et al., 2008).

The effector GobX is the second U-box E3 ligase encoded by *L. pneumophila*, this enzyme is *S*-palmitoylated in host cells, thus allowing it to specifically target to the Golgi apparatus (Lin et al., 2015). Although its substrate has not yet been identified, such localization suggests that GobX functions to modulate cellular activity associated with this organelle (Lin et al., 2015). Recently, Lin et al. (2018) reported the amino terminal portion of RavN (Lpg1111) contains a U-box-like motif that has E3 activity. These authors also found that HipA (Lpg2370), MavM (Lpg2577), and MavJ (Lpg2498) have E3 ligase activity despite the fact that they share very limited homology to eukaryotic E3s. Unfortunately, the substrates of these E3s are currently unknown, so is their biological significance in *L. pneumophila* infection.

The SCF complex is a multi-subunit ubiquitin ligase consisting of RING-Box1 (RBXI), cullin 1 (CUL1), S-phase kinase-associated protein 1 (SPK1) and an F-box motif containing protein (FBP) (Nguyen and Busino, 2020). Whereas the first three proteins serve as structural scaffold for the complex to interact with other proteins, particularly an appropriate E2 enzyme, FBP contributes to substrate specificity of the SCF complex by modules involved in protein-protein interaction such as leucine-rich repeats and WD repeats (Nguyen and Busino, 2020). The *L. pneumophila* strain Philadelphia 1 encodes at least 5 FBP-like effectors to engage ubiquitination (Ensminger and Isberg, 2010; Skaar et al., 2013). Among these, LegU1 (Lpg0171) and AnkB (Lpg2144) contain *bona fide* F-box motifs and have been demonstrated to form active SCF complexes by interacting with SKP1. LicA (Lpg1408) harbors an F-box motif and this protein interacts with SKP1 but does not detectably bind CUL1 (Ensminger and Isberg, 2010). The E3 ligase complex formed by LegU1 catalyzes ubiquitination of the HLAB-associated transcript-3 (BAT3) (Ensminger and Isberg, 2010), a protein involved in the regulation of a wide range of cellular processes, including apoptosis in higher eukaryotes (Sasaki et al., 2007; Corduan et al., 2009). By a yeast two-hybrid screen, Lomma et al. (2010) identified ParvB as a substrate for AnkB and found that the bacterial F-box protein functions to reduce ParvB ubiquitination induced by endogenous enzymes. Yet whether ParvB is a specific substrate ubiquitylated by AnkB needs further study. AnkB is required for optimal intracellular growth of *L. pneumophila* strains AA100 and Paris in human monocyte derived macrophages (hMDMs) and for lung colonization in A/J mice (Price et al., 2009; Lomma et al., 2010). However, AnkB is dispensable for intracellular replication of strain Paris in *Acanthamoeba castellanii* (Lomma et al., 2010), while it is required for proficient replication of strain AA100 in *A. polyphaga* (Al-Khodor et al., 2008). These discrepancies may be due to differential effector redundancy in different strains of *L. pneumophila* or/and different protozoa infection models used. Finally, although both PpgA (Lpg2224) and Lpg2525 are predicted to harbor an F-box by bioinformatic analysis, neither has been shown to interact with SKP1 (Ensminger and Isberg, 2010). Whether these proteins have E3 ligase activity remained to be determined.

## SidC and SdcA

One prominent feature of the LCV is that this organelle dynamically recruits and retains an array of proteins of either host or *L. pneumophila* origin. In some cases, the anchoring of bacterial proteins on the LCV is mediated by their binding to phosphatidylinositol-4-phosphate (PI4P), an important signaling lipid whose enrichment on the bacterial phagosome is achieved in part by sequential action of three independent effectors that coordinate to biosynthesize PI4P from phosphatidylinositol (Hsu et al., 2012; Dong et al., 2016; Li et al., 2021). SidC (Lpg2511) and its ortholog SdcA anchor on the LCV by binding to PI4P via a lipid-interacting domain localized in the carboxyl portion of these proteins (Weber et al., 2006; Ragaz et al., 2008; Luo et al., 2015). These two highly similar effectors function to facilitate the recruitment of ER-derived vesicles and ubiquitylated proteins to the LCV, which is executed by an activity conferred by their amino terminal domain (Ragaz et al., 2008), a region later found to possess E3 ligase activity (Hsu et al., 2014). The biochemical basis of ubiquitination catalyzed by SidC and SdcA is unique, which requires a Cys-His-Asp catalytic triad (Hsu et al., 2014), which often is associated with proteases (Buller and Townsend, 2013). Although these two proteins are of 72% identical in their primary sequences, SidC prefers the E2 UbcH7 for its activity and predominantly catalyzes the formation of K11- and K33-linked polyubiquitin chains. In contrast, SdcA displays the highest activity when working together with the E2 UbcH5 (Hsu et al., 2014). The E3 ligase activity of SidC and SdcA is essential for their role in the recruitment of ER components to the LCV (Hsu et al., 2014; Luo et al., 2015). Intriguingly, structural analysis reveals that the four α-helix bundles of the PI4P-binding motif blocks the catalytic site for the E3 activity embedded in the SNL (SidC N-terminal ubiquitin ligase) domain (Luo et al., 2015). In biochemical reactions, truncation mutants lacking the PI4P-binding domain exhibits more robust E3 ligase activity than the full-length protein. Consistently, inclusion of PI(4)P in reactions with full-length SidC enhances its E3 ligase activity (Luo et al., 2015). Thus, SidC and SdcA become more active in the presence of PI(4)P, which may allow them to selectively ubiquitinate their substrates on the surface of the LCV. Interestingly, Lpg2452 (SdcB) also possess a domain structurally resembling the catalytic center of SidC important for its E3 ligase activity, thus had been included as a member of the SidC family (Lin et al., 2018).

The small GTPase Rab1 is monoubiquitinated in macrophages infected with wild-type *L. pneumophila* but not the Δ*sidCsdcA* mutant, suggesting that Rab1 is a substrate of these E3 enzymes (Horenkamp et al., 2014). However, ubiquitination of Rab1 by SidC or SdcA was not detected in cells co-expressing these proteins by transfection or in biochemical reactions with recombinant proteins (Horenkamp et al., 2014; Hsu et al., 2014). How infection of wild-type *L. pneumophila* induces Rab1 ubiquitination remains to be investigated. Recently, Jeng et al. (2019) identified the GTPase Rab10 as a substrate of SidC and SdcA. Consistent with the non-degradative K11- and K33-types of polyubiquitin chains induced by SidC and SdcA, Rab10 is not degraded by SidC or SdcA. Instead, ubiquitination leads to its recruitment to the LCV (Jeng et al., 2019). How ubiquitination of Rab10 and its recruitment to the bacterial phagosome benefit the bacterium is unknown.

## Dubs

Ubiquitination can be reversed by enzymes of the deubiquitinase superfamily. Members of this large family of proteases function to cleave Ub from ubiquitinated substrates by hydrolyzing the isopeptide bond between Ub moieties and between Ub and the substrate (Harrigan et al., 2018). The specificity of Dubs toward their substrates is dictated by multiple factors, including their topography, the polyubiquitin chain types, and the way the enzyme makes contact with Ub moieties (Ronau et al., 2016). *L. pneumophila* codes for multiple Dubs that participate in various aspects of its interactions with host cells (Kitao et al., 2020a). Among these, LupA (Lpg1148) is the first described Dub in this pathogen, it harbors a Cys-His-Asp catalytic triad associated with typical bacterial CE clan Dubs (Urbanus et al., 2016). LupA is able to suppress the yeast toxicity of LegC3 (Lpg1701), another Dot/Icm substrate that appears to modulate membrane fusion in host cells (Urbanus et al., 2016). LegC3 undergoes ubiquitination in host cells probably by a yet unidentified host E3 ligase and LupA can remove such modification in a process that requires an intact Cys-His-Asp catalytic triad (Urbanus et al., 2016). How the Dub activity of LupA suppresses the yeast toxicity of LegC3 is not fully understood. One possibility is that ubiquitination activates the enzymatic activity of LegC3, which causes yeast toxicity and the removal of the Ub by LupA thus will put the toxicity under check.

LotA (Lpg2248) harbors two ovarian tumor (OTU)-like domains each possessing its own catalytic cysteine residue (Cys13 and Cys303, respectively). Whereas the domain using Cys13 mainly cleaves Lys6-linked polyubiquitin chains, the other prefers Lys48/Lys63-linked polyubiquitin (Kubori et al., 2018). Although its target is unknown, LotA contributes to the removal of ubiquitinated proteins from the surface of the LCV and is required for proficient intracellular growth of *L. pneumophila* (Kubori et al., 2018). LotB (Lpg1621) is another OTU-like Dub that specifically removes Lys63-linked ubiquitin chains (Ma et al., 2020; Schubert et al., 2020; Shin et al., 2020a). Structural analysis reveals that except for the ubiquitin binding site S1, LotB harbors an additional site S1’, which confers its specificity in cleavage of the Lys63-linked chains (Shin et al., 2020a). The SNARE protein Sec22b involved in membrane fusion is a substrate of LotB; this protein is ubiquitinated in cells infected with *L. pneumophila* and LotB appears to function to counteract such modification (Shin et al., 2020a). The removal of ubiquitin from Sec22b abolishes the interaction between Sec22b on the LCV and the t-SNARE syntaxin 3, resulting in the dissociation of the latter from the bacterial phagosome (Kitao et al., 2020b). Interestingly, ubiquitination and recruitment of Rab10 to the LCV by SidC and SdcA are regulated by a third OUT-like Dub Lem27 (Lpg2529) (also known as LotC) coded for by *L. pneumophila* (Schubert et al., 2020; Shin et al., 2020a). Lem27 appears to modulate Rab10 ubiquitination by counteracting the activity of SidC and SdcA (Liu et al., 2020). Ceg7 (Lpg0227) has also been shown to possess Dub activity and belongs to the OUT-like family (Hermanns et al., 2020; Schubert et al., 2020). This enzyme broadly targets K6-, K11-, K48-, and K63-linked polyubiquitin chains in biochemical assays but the biological significance of this activity is unclear (Schubert et al., 2020). A recent structural study revealed that an extended helical lobe (EHL) domain commonly shared in LotA, LotB and Lem27 is responsible for their Ub binding, defining the Lot-OTUs as a unique class of Dubs (Takekawa et al., 2021).

RavD (Lpg0160) is an effector that is associated with the LCV by binding to phosphatidylinositol-3-phosphate (PI3P) (Pike et al., 2019). Although no impact on bacterial intracellular growth, deletion of *ravD* led to a significant increase of late endosome/lysosome markers such as LAMP-1 on the LCV (Pike et al., 2019). A more recent study revealed that RavD is a Dub that specifically cleaves linear ubiquitin chains (Wan et al., 2019b), a unique ubiquitin chain important for signaling in innate and adaptive immunity, and inflammatory signaling (Rittinger and Ikeda, 2017). The removal of the linear ubiquitin chains on the LCV by RavD benefits *L. pneumophila* by suppressing host immune responses (Wan et al., 2019b). The activity of RavD suggests *L. pneumophila* may more extensively interact with higher order hosts, including mammals, because the ubiquitin ligase complex LUBAC responsible for the formation of linear ubiquitin chain is not present in protozoan (Spit et al., 2019).

## Phosphoribosyl Ubiquitination and Its Regulation

**FIGURE 2:**
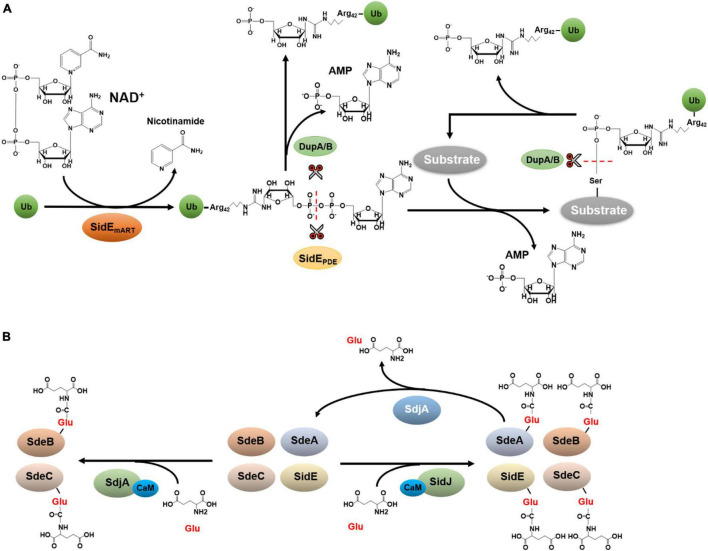
Ubiquitination by the SidE family and its regulation. **(A)** Phosphoribosyl ubiquitination and de-ubiquitination. Phosphoribosyl ubiquitination induced by SidEs is initiated by ADP-ribosylation of Ub at Arg42 by its mART activity, leading to the release of nicotinamide and the production of ADPR-Ub. The phosphodiester bond in ADPR-Ub is subsequently the cleaved by the phosphodiesterase (PDE) domain of SidEs, which is accompanied by the transfer of phosphoribosyl Ub to serine residues of substrate proteins and the release of AMP. **(B)** Glutamylation and de-glutamylation of members of the SidE family. In host cells, SidJ binds to CaM via an IQ motif in its carboxyl end. The complex functions as a glutamylase to attack the first glutamate residue of the ExE motif in the mART domain of SidEs by polyglutamylation, leading to abolishment of the activity of the mART, and thus the inactivation of Ub ligase activity of the SidEs. Despite its high-level similarity to SidJ, SdjA selectively inhibits SdeB and SdeC by glutamylation. In addition to the glutamylase activity, SdjA also exhibits a deglutamylase activity toward glutamylated SdeA.

The SidE family contains SdeA, SdeB, SdeC, and SidE, which are four large proteins. Among these, SidE, SdeA, and SdeC are almost identical at the primary sequence level, only with a few conserved substitutions throughout the proteins of about 1,500 residues. SdeB (1926 aa) is considerably larger than the other three members of the family, it contains an extra region of about 400 aa in its carboxyl end (Luo and Isberg, 2004). Members of the SidE family (SidEs) are functionally redundant in *L. pneumophila* virulence (Bardill et al., 2005). Careful biochemical analysis reveals that these effectors represent a novel Ub ligase that catalyzes ubiquitination by a mechanism that is chemically distinct from the canonical three-enzyme cascade. First, Ub activation in reaction catalyzed by SidEs is energized by nicotinamide adenine dinucleotide (NAD), which is hydrolyzed by a mono-ADP-ribosyltransferase activity (mART) to modify Arg42 of Ub to produce ADP-ribosylated Ub (ADPR-Ub) (Qiu et al., 2016; Figure 2A). Whereas the biochemical basis of this catalysis is similar to classical mART often found in bacterial toxins (Mikolcevic et al., 2021), ADPR-Ub produced by this reaction is utilized by a phosphodiesterase (PDE) activity also embedded in each of SidE family member, which cleaves the phosphoanhydride bond between the two phosphate atoms, resulting in the transfer of phosphoribosyl Ub (PR-Ub) to serine residues on substrate proteins and the release of AMP (Bhogaraju et al., 2016; Kotewicz et al., 2017; Figure 2A).

Members of the SidE family appear to attack a large cohort of structurally diverse host proteins, including a number of Rab small GTPases and numerous ER resident proteins such as Rtn4 and FAM134C (Bhogaraju et al., 2016; Qiu et al., 2016; Kotewicz et al., 2017; Wan et al., 2019a; Shin et al., 2020b). Although many of these substrates are similarly attacked by each of these ligases (Bhogaraju et al., 2016; Qiu et al., 2016; Kotewicz et al., 2017; Wan et al., 2019a; Shin et al., 2020b), it is not clear whether any of these proteins specifically modifies one or more host proteins. Structural analysis suggests that a binding cleft in the PDE domain is responsible for substrate recognition which engages a sequence motif in which the target serine is in the vicinity of hydrophobic residues and is flanked by proline residues (Kalayil et al., 2018).

During *L. pneumophila* infection, ubiquitination of Rtn4 by SidEs drastically promotes structural transformation of ER tubules, probably by inducing Rtn4 oligomerization, thus providing a scaffold for the formation of tubule matrix-like structures (Kotewicz et al., 2017). For small GTPases such as Rab33b, modification induced by SidEs leads to slight inhibition of its GTPase activity (Qiu et al., 2016). The modification and subsequent recruitment of other ER proteins such as FAM134C to the LCV may prevent the delivery of the bacterial phagosome to the autophagic pathway (Wan et al., 2019a; Shin et al., 2020b). Phosphoribosyl ubiquitination of Rab33b by SidEs recruits this GTPases to the LCV, where its associates with another small GTPase Rab6A, which interacts with the ER via direct interaction with SNAREs. Thus, one role of the SidE family is to promote the fusion between the LCV and the ER (Kawabata et al., 2021).

Members of SidEs family also have classical Dub activity, each conferred by a domain formed by the first 200 residues of the proteins (Sheedlo et al., 2015). The cleavage of isopeptide bond by these Dub domains is catalyzed by a Cys-His-Asp triad commonly found in the CE clan proteases (Sheedlo et al., 2015). This Dub module cleaves Lys11, Lys48, and Lys63-linked polyubiquitin chains, with a distinct preference for Lys63 linkages (Sheedlo et al., 2015). The Dub activity of SidEs function to reduce the association of ubiquitinated proteins on the LCV, which may antagonize the recruitment of host autophagy machinery to the phagosome. In addition, the low chain type specificity of these Dub domains probably function to replenish the Ub pool proximal to the LCV to provide the reaction precursor for phosphoribosyl ubiquitination.

Similar to canonical ubiquitination, modification by SidEs is reversible. The phospho-ribose linkage between Ub and the substrate cannot be cleaved by canonical Dubs. By testing a number of *Legionella* proteins predicted to harbor a PDE domain potentially involved in cleaving phosphodiester bond, two independent studies identified DupA (Lpg2154) and DupB (Lpg2509) as enzymes that function to remove PR-Ub from substrates (Akturk et al., 2018; Wan et al., 2019a; Figure 2A). Intriguingly, the Dup family can hydrolyze ADPR-Ub to produce PR-Ub and AMP (Akturk et al., 2018; Wan et al., 2019a; Figure 2A).

Structural studies reveal that the distance between the mART domain of SidEs and the PDE domain is approximately 70 Å (Akturk et al., 2018; Dong et al., 2018; Kalayil et al., 2018), suggesting the need of a mechanism to channel the reaction intermediate ADPR-Ub produced by the mART domain to the second reaction center. Because ADPR-Ub is highly toxic to eukaryotic cells by interfering with canonical ubiquitination (Bhogaraju et al., 2016), hydrolysis of this reaction intermediate by DupA and DupB may prevent poisoning of the host cell by ADPR-Ub that accidentally escapes from the mART catalytic pocket. Whether and how PR-Ub, which is also toxic to eukaryotic cells (Bhogaraju et al., 2016), is further reduced to phosphoribose and free Ub is unknown.

In addition to DupA and DupB, which mainly function to reverse ubiquitination induced by SidEs, the activity of this family of Ub ligases is further regulated by SidJ, one protein of a two-member family (Liu and Luo, 2007). SidJ had been reported to disperse SidEs from the LCV (Jeong et al., 2015) and can suppress the toxicity of SidEs to eukaryotic cells (Havey and Roy, 2015; Jeong et al., 2015). Of note is that the regulation of cellular distribution of SidEs by SidJ was not detected in an independent study (Qiu et al., 2017). Four structural and biochemical studies independently reveal that SidJ is a glutamylase that attacks the first glutamate residue of the ExE motif in the mART domain of SidEs by polyglutamylation (Bhogaraju et al., 2019; Black et al., 2019; Gan et al., 2019b; Sulpizio et al., 2019). This modification abolishes the activity of the mART, thus the Ub ligase activity of the SidEs. Mechanistically, the glutamylation by SidJ is catalyzed by a two-step reaction in which the enzyme first activates the target glutamate residue by acyl-adenylation using a pseudokinase domain (Sreelatha et al., 2018), followed by the replacement of the AMP moiety with glutamate (Bhogaraju et al., 2019; Black et al., 2019; Gan et al., 2019b; Sulpizio et al., 2019), Interestingly, SidJ is activated by the eukaryote-specific calcium-binding protein calmodulin (CaM) which recognizes an IQ motif in its carboxyl end (Bhogaraju et al., 2019; Black et al., 2019; Gan et al., 2019b; Sulpizio et al., 2019). The requirement of CaM restricts the activity of SidJ in the cytosol of the infected cell, thus preventing premature inactivation of SidEs. Despite its high-level similarity to SidJ, SdjA, the other member of the SidJ family, cannot rescue the yeast growth defect caused by SdeA (Qiu et al., 2017; Gan et al., 2019b). Furthermore, SidJ and SdjA are not functionally redundant for the intracellular growth defect of the Δ*sidJ* mutant cannot be complemented by SdjA (Liu and Luo, 2007). A recent study found that SdjA selectively inhibits some members of the SidE family by glutamylation (Osinski et al., 2021; Figure 2B). Interestingly, another study shows that in addition to the glutamylase activity against SdeB and SdeC, SdjA exhibits a deglutamylase activity toward SdeA that has been modified by SidJ/CaM (Song et al., 2021; Figure 2B). Thus, SdjA functions to fine-tune the activity of SidJ and SdeA during *L. pneumophila* infection (Song et al., 2021). Yet, the mechanism by which SdjA catalyzes the cleavage of the isopeptide bond between glutamate residues in glutamylated SdeA needs further investigation.

The SidE effector family has long known to be important for optimal virulence of *L. pneumophila* (Song et al., 2021), and targeting the unique ubiquitin ligase activity with compounds is an anti-virulence strategy to control *L. pneumophila* infection. Several stable NAD^+^ analogs capable of inhibiting the activity of SdeC at IC_50_ of 28–39 μM have been developed (Madern et al., 2020). It remains unclear whether these compounds can block intracellular replication of the pathogen.

## Reversible Ubiquitination by Transglutaminases MavC and MvcA

**FIGURE 3:**
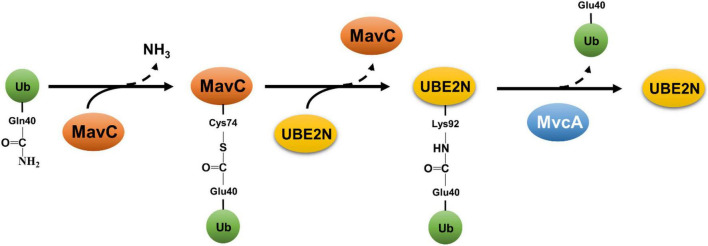
Reversible atypical ubiquitination by MavC and MvcA. Ubiquitination catalyzed by MavC is initiated by the formation of a thioester linkage between the active residue Cys74 and Gln40 of Ub, concomitant with the release of ammonium. The acylated MavC then reacts with the amine donor from a lysine (Lys92) in UBE2N to form an intermolecular isopeptide bond, resulting in ubiquitination of UBE2N. MvcA, a structural ortholog of MavC, functions as a deubiquitinase to cleave the isopeptide bond between Ub and UBE2N catalyzed by MavC, leading to the release of UBE2N and deamidated Ub.

The second non-canonical ubiquitination mechanism by *L. pneumophila* is exemplified by MavC (Lpg2147) that catalyzes a crosslink reaction between Ub and the E2 enzyme UBE2N (Gan et al., 2019a). MavC is a transglutaminase that induces the formation of a γ-glutamyl-ε-Lys isopeptide between Gln40 of Ub and Lys92 of UBE2N (Gan et al., 2019a). This reaction appears to preferably occur intramolecularly in charged UBE2N that carries a Ub moiety covalently linked to its active site Cys87 (Puvar et al., 2020). The reaction catalyzed by MavC is initiated by the formation of a thioester linkage between its active site Cys74 and the side chain amide of Gln40 of Ub, which is followed by a transglutamination reaction that leads to the installation of an isopeptide bond between Lys92 of UBE2N and Gln40 of Ub (Guan et al., 2020; Puvar et al., 2020; Figure 3). Because of its structural homology to bacterial deamidases Cif and CHBP that attack Ub and NEDD8 at Gln40 (Cui et al., 2010), MavC was identified in an earlier study as a Ub deamidase (Valleau et al., 2018). All described transglutaminases exhibit a protein deamidation activity in reactions lacking the crosslink target (Lorand and Graham, 2003). Consistent with this notion, MavC deamidates Ub at Gln_40_ in reactions containing only the enzyme and Ub (Valleau et al., 2018; Gan et al., 2019a). Importantly, the Ub deamidation activity is only detectable in biochemical reactions with large amounts of proteins but not in cells infected with wild-type *L. pneumophila* (Valleau et al., 2018; Gan et al., 2019a). Structurally, the main domain of MavC resembles the structures of Cif effectors (Valleau et al., 2018), the insertion domain is involved in engaging its substrate UBE2N (Guan et al., 2020).

MvcA (Lpg2148) is an ortholog of MavC, these two proteins share 50% identity and 65% similarity in their sequences and the structures of these two proteins are superimposable (Cui et al., 2010). Both MavC and MvcA deamidates Ub at Gln40, and this activity had been suggested to interfere with Ub signaling at different phases of *L. pneumophila* infection (Cui et al., 2010). Despite this high-level similarity, MvcA cannot catalyze protein crosslink between Ub and UBE2N or several other E2 enzymes (Gan et al., 2020). Further biochemical experiments reveal that MvcA functions as deubiquitinase that cleaves the isopeptide bond between Ub and UBE2N in the crosslink product formed by MavC (Gan et al., 2020; Mu et al., 2020; Figure 3). MavC and MvcA utilize the same biochemical mechanism to catalyze two reactions that have an opposite effect toward the activity of UBE2N (Gan et al., 2020; Mu et al., 2020). It has been proposed that the outcome of the reaction is determined by the binding affinity of the enzyme to Ub, UBE2N and the product UBE2N-Ub (Guan et al., 2020). The surface charge of the Tail domain of MvcA is repulsive to Ub, which together with the lower binding affinity for UBE2N favor the dissociation of Ub from MvcA, thus resulting in the cleavage of the isopeptide bond (Guan et al., 2020).

A main function of UBE2N is to catalyze the formation of K63-type polyubiquitin chains, which are involved in the regulation of diverse cellular activities, including immunity by inducing the activation of the NFκB pathway (Hodge et al., 2016). MavC-induced ubiquitination UBE2N inserts the Ub moiety in the catalytic pocket of the E2 enzyme (Gan et al., 2020; Mu et al., 2020), which blocks the entry of activated Ub to its Cys87 active site. Although MavC can effectively induce crosslink between free Ub and UBE2N, charged UBE2N carrying a Ub moiety on its catalytic site Cys87 via a thioester bond appears to be the prefer substrate (Puvar et al., 2020). This modification effectively inhibits NFκB activation by cues that initiate the signaling upstream of UBE2N (Valleau et al., 2018; Gan et al., 2019a). Like many Dot/Icm effectors, MavC expresses at high levels in bacteria grown to the post-exponential phase (Gan et al., 2019a), which facilitates the initial attack of the host defense upon contact. As the infection proceeds to later phases, the activity of UBE2N is restored by MvcA to accommodate the need of NFκB activation for maintaining critical cellular functions such as cell survival (Losick and Isberg, 2006). Consistent with this notion, the expression of MvcA does not become detectable several hours after bacterial uptake (Gan et al., 2020). Interestingly, Lpg2149 which is encoded by a gene in the *mavC* and *mvcA* locus, blocks the activity of both MavC and MvcA by direct binding (Mu et al., 2020). The biological significance of Lpg2149 largely remains mysterious, but its presence further highlights the importance of fine-tuning the activity of UBE2N during *L. pneumophila* infection.

## Conclusion and Perspectives

The study of *L. pneumophila* effectors involved in ubiquitination has greatly expanded our appreciation of the exploitation of Ub signaling by a pathogen. It has also extended our understanding of the chemical basis of ubiquitination. A recent study shows that Ub signaling plays important roles beyond the remodeling of phagosomal membranes (Ong et al., 2021). Several host proteins involved in ubiquitination, particularly the E2 enzyme UBE2E1, and the E3 Ub ligase, CUL7, are important not only for *L. pneumophila* intracellular replication but also for Dot/Icm-mediated protein translocation (Ong et al., 2021). Clearly, the involvement of the Ub system in *L. pneumophila* pathogenesis is deeper and more extensive than we have appreciated. It will be of great interest to determine whether and how these host enzymes impact the functionality of the bacterial protein translocation machinery and its substrates. An equally important question is whether these enzymes are targeted by *L. pneumophila* effectors involved in Ub signaling.

With a few exceptions, the cellular targets of most of the E3 ligases or Dubs remain unknown. Clearly, the identification of their substrates is essential in our understanding of these effectors in the intracellular life cycle of *L. pneumophila.* Because the affinity between E3 ligases and their substrates often is too low to be harnessed for substrate identification (Nagy and Dikic, 2010), new technologies such as orthogonal Ub transfer (OUT) (Zhao et al., 2012), effective proximity labeling by the bacterial biotin ligase BirA (Branon et al., 2018) and substrate trapping by fusion proteins (O’Connor et al., 2015), will surely be useful in this endeavor. It is anticipated that more exciting discoveries will be made in years to come in our investigation of Ub signaling in *L. pneumophila* virulence.

## Author Contributions

JL and LW drafted the first version of the manuscript. LS and Z-QL revised the manuscript with input from all authors.

## Conflict of Interest

The authors declare that the research was conducted in the absence of any commercial or financial relationships that could be construed as a potential conflict of interest.

## Publisher’s Note

All claims expressed in this article are solely those of the authors and do not necessarily represent those of their affiliated organizations, or those of the publisher, the editors and the reviewers. Any product that may be evaluated in this article, or claim that may be made by its manufacturer, is not guaranteed or endorsed by the publisher.
